# A Proof-of-Principle Study of Non-invasive Identification of Peanut Genotypes and Nematode Resistance Using Raman Spectroscopy

**DOI:** 10.3389/fpls.2021.664243

**Published:** 2022-01-04

**Authors:** William Z. Payne, Tianyi Dou, John M. Cason, Charles E. Simpson, Bill McCutchen, Mark D. Burow, Dmitry Kurouski

**Affiliations:** ^1^Department of Biochemistry and Biophysics, Texas A&M University, College Station, TX, United States; ^2^Texas A&M AgriLife Research, Stephenville, TX, United States; ^3^Department of Plant and Soil Science, Texas Tech University, Lubbock, TX, United States; ^4^Texas A&M AgriLife Research, Lubbock, TX, United States; ^5^The Institute for Quantum Science and Engineering, Texas A&M University, College Station, TX, United States

**Keywords:** peanut varieties, Raman spectroscopy, phenotyping, identification, genotyping, nematode resistance

## Abstract

Identification of peanut cultivars for distinct phenotypic or genotypic traits whether using visual characterization or laboratory analysis requires substantial expertise, time, and resources. A less subjective and more precise method is needed for identification of peanut germplasm throughout the value chain. In this proof-of-principle study, the accuracy of Raman spectroscopy (RS), a non-invasive, non-destructive technique, in peanut phenotyping and identification is explored. We show that RS can be used for highly accurate peanut phenotyping *via* surface scans of peanut leaves and the resulting chemometric analysis: On average 94% accuracy in identification of peanut cultivars and breeding lines was achieved. Our results also suggest that RS can be used for highly accurate determination of nematode resistance and susceptibility of those breeding lines and cultivars. Specifically, nematode-resistant peanut cultivars can be identified with 92% accuracy, whereas susceptible breeding lines were identified with 81% accuracy. Finally, RS revealed substantial differences in biochemical composition between resistant and susceptible peanut cultivars. We found that resistant cultivars exhibit substantially higher carotenoid content compared to the susceptible breeding lines. The results of this study show that RS can be used for quick, accurate, and non-invasive identification of genotype, nematode resistance, and nutrient content. Armed with this knowledge, the peanut industry can utilize Raman spectroscopy for expedited breeding to increase yields, nutrition, and maintaining purity levels of cultivars following release.

## HIGHLIGHTS

- We show that Raman spectroscopy can be used for highly accurate identification of nematode resistance and susceptibility in peanuts. This allows for the use of Raman in digital selection of plant species.

## Introduction

The population of the world is increasing at an alarming rate. As population increases, the production of food must increase in order to match demand. It is predicted that we will need to produce 70% more food by 2050 to sustain our population (Food and Agriculture Organization of the United Nations, 2009). As population increases, the expansion of agricultural territory is limited due to urbanization, cost of production, and other contributing factors. The continued loss of agricultural lands has led agricultural leaders to focus on increasing yields on existing croplands through the innovation of digital farming. Digital farming, also referred to as precision agriculture, seeks to maximize crop yield by maximizing plant production and minimizing the environmental impact with the use of technologies such as Raman spectroscopy (Farber et al., 2019a, 2020b; Sanchez et al., 2019a,b, 2020b).

Although many practices in modern agriculture have reached a significant degree of mechanization, genotyping and taxonomic identification for the purpose of plant breeding is not one of them. Identity preservation and seed purity is a growing problem as more and more value-added traits are incorporated into new varieties. Similar issues are faced by plant breeders as well as seedsmen and processors. Historically, plant descriptors and a “trained eye” have been used to identify cultivars and maintain seed purity. More recently, genotyping techniques, whether it be marker-assisted selection (MAS) (Chu et al., 2011; Burow et al., 2013, 2019) to select for specific traits of interest, or genomic selection (GS) (Hayes et al., 2009; Heffner et al., 2009; Ravelombola et al., 2019, 2020), which can be used to identify elite breeding materials, have been used to develop cultivars with beneficial traits and move them into production. A trained eye requires an expert with substantial taxonomic knowledge and many years of experience. Even with an expert as described, visual inspection is subjective and often difficult even for those with bountiful experience. Genotyping, whether by sequencing or other methods, is more accurate than visual inspection. However, genotyping has its drawbacks. In the early stages of cultivar development, seed can be very limited in a crop such as peanut. In these early generations, everything must be handled by hand which is time-consuming and labor-intensive but makes genotyping feasible. However, as one move into larger and larger lots, genotyping becomes impractical on a large scale where profit margins are small. Anything that can lessen these burdens during the development process would represent a significant improvement.

Raman spectroscopy (RS) is a label-free, non-invasive, and non-destructive analytical technique that can be used to examine the chemical composition of samples (Farber et al., 2019a, 2020c). The Kurouski group recently demonstrated that RS can be used to detect both biotic and abiotic stress on plants. Using a hand-held Raman spectrometer, the Kurouski group showed one can diagnose fungal disease in corn, wheat, or sorghum with great accuracy (Egging et al., 2018; Farber and Kurouski, 2018). Another study done by the Kurouski lab showed that RS could be used to identify potato variety, origin of cultivation, and starch content (Morey et al., 2020). These studies demonstrate RS’s usefulness in detecting changes in plants and the potential to revolutionize agriculture.

Peanut (*Arachis hypogaea* L.) is an allotetraploid (2n = 4x = 40) that has been cultivated for thousands of years (Singh and Simpson, 1994). Today, peanut is grown throughout the temperate and tropical parts of the world (Kochert et al., 1991; Krapovickas and Gregory, 1994, 2007). In the U.S., approximately 556,000 ha of peanuts were harvested in 2018, with an average yield of 4,484 kg/ha (USDA-NASS, 2019). The estimated farm value of U.S. production in 2019 was approximately $1.2 billion, resulting in peanut being the third most valuable cash crop based on net revenue (USDA-NASS, 2019). Peanuts are used in many popular food products in the U.S. which results in Americans consuming, on average, more than 6 pounds of peanut products a year and spending over 2 billion dollars on peanut products at the retail level (NPB, 2020).

A major pest associated with peanut production is the root-knot nematode [*Meloidogyne arenaria* (Neal)]. Root-knot nematodes are found throughout the peanut production regions in the U.S. from Georgia to Texas. The peanut root-knot nematode can cause significant yield losses (Tirumalaraja et al., 2011). It has been estimated that yield losses of 3–15% are common (Dong et al., 2007) and heavily infested fields can see losses of 75% or more (Rich and Tillman, 2009). Peanut suffers from a narrow genetic base (Kochert et al., 1991) and no longer has access to many genes contained in related wild relatives. However, many genes associated with both biotic and abiotic stressors have been identified (Cason et al., 2020), and gene introgression has successfully been used to move alleles into cultivated peanut and is still one of the best options available to peanut breeders (Cason et al., 2020). An excellent example of this is the resistance to root knot transferred from *Arachis cardenasii* (Burow et al., 1996; Simpson et al., 2003). The resulting introgression resulted in almost total immunity to root-knot nematode (Simpson and Starr, 2001; Simpson et al., 2003, 2013). While not fully understood, it is believed the resistance is associated with a failure by juveniles to establish a feeding site that causes root-knot nematode resistance (Timper et al., 2000). This has resulted in the release of the resistant cultivars: COAN (Simpson and Starr, 2001), NemaTAM (Simpson et al., 2003), Webb (Simpson et al., 2013), Georgia 14N (Branch and Brenneman, 2014), Tifquard (Holbrook et al., 2008), and TifNV High O/L (Holbrook et al., 2017).

In this proof-of-principle study, we demonstrate that RS can be used for highly accurate identification of peanut genotypes based on spectroscopic analysis of their leaves. We also show that RS can further be used to screen these peanut leaves for the identification of specific traits in germplasm, such as nematode resistance, based on the direct analysis of biochemical profile of the leaves which identify molecular species that are unique to nematode-resistant germplasm.

## Materials and Methods

### Peanut Germplasm

Approximately 30 leaves from 20 different genotypes of peanut (see Table 1) were provided by the Texas A&M AgriLife Research and Extension Center at Stephenville. The plants were grown as part of the Texas A&M Peanut Breeding Programs Statewide Advanced Line Breeding Yield Trial in Erath Co., Texas. The trial was planted on May 7, 2020 and managed according to recommended production practices. The trial was designed with a randomized complete block design (RCBD) containing 16 breeding lines and 4 commercially available checks. Each experimental unit was 3 × 3 m in two row plots replicated three times. For this project, plots were sampled on September 17, 2020. Individual leaves were sampled randomly from the lateral branches within each plot and bulked by plot. These peanut leaves were scanned approximately twice per leaf depending on the size of the leaf.

**TABLE 1:** A complete list of varieties and breeding lines (genotypes) in the 2020 Advance Line Trial from Erath Co., Texas.

Entry	Genotype	Entry	Genotype	Entry	Genotype	Entry	Genotype
1	Tx121082	6	Tx200606-2-11	11	TP200610-1-14	16	TP200610-4-8
2	Tx144342	7	TxL100212-03-03	12	TxL100212-05-09	17	Webb
3	Tx144370	8	TP200606-3-3	13	TxL100212-07-07	18	Tamrun OL11
4	Tx144485	9	TP200609-1-5	14	TP200610-2-9	19	Georgia 09B
5	TxL100212-02-05	10	TxL100225-03-13	15	TP200610-3-7	20	Georgia 14N

### Raman Spectroscopy

#### Acquisition

A portable, hand-held Agilent Resolve spectrometer with an 830 nm laser equipped was used to collect all spectra. The experimental parameters used for the collected spectra were: 1 s acquisition time, 495 mW power, and surface scan mode. Leaves were gently pressed against the nose cone for proper focus during scans. Not all leaves that were provided resulted in usable scans, so in some cases samples were bulked to allow for a number of scans that was consistent. In total, we collected over 1,200 spectra from leaves of both nematode-resistant peanut plants and non-nematode-resistant peanut plants.

#### Processing

Spectra were automatically baseline-corrected and background subtracted by the onboard software of the instrument. Data from the instrument were exported as comma separated value (CSV) files using provided software from the company. These CSV’s were imported into MATLAB for preprocessing. Statistical analysis of spectra was than conducted using PLS_Toolbox, an add-on of MATLAB.

### Statistical Analysis

#### Partial Least Squares Discriminant Analysis

Spectra were imported into MATLAB for multivariate statistical analysis. Partial least squares discriminant analysis (PLS-DA) was used to build classification models. PLS-DA, an extension of ordinary PLS, uses dummy Y-variables to indicate discrete classes/categories of data which the model then proceeds to predict (Eriksson et al., 2013). PLS-DA is a type of supervised learning model and the user must provide categories during training for each data point. After the model finishes training, it then cross-validates. This means part of the dataset is excluded while the rest is used to train the model. The model tries to predict the class membership of the excluded data points. This process repeats until all data points have been included. In this study, cross-validation results are reported and are suggestive of the model’s ability to classify unseen data. Differentiation of peanut varieties using leaf spectra were conducted in the MATLAB add-on PLS_Toolbox using PLS-DA. The selected preprocessing used for modeling included: SNV, 1st derivative, smoothing, PQN, normalize, and mean center.

## Results and Discussion

### Differentiation of Genotype

**FIGURE 1:**
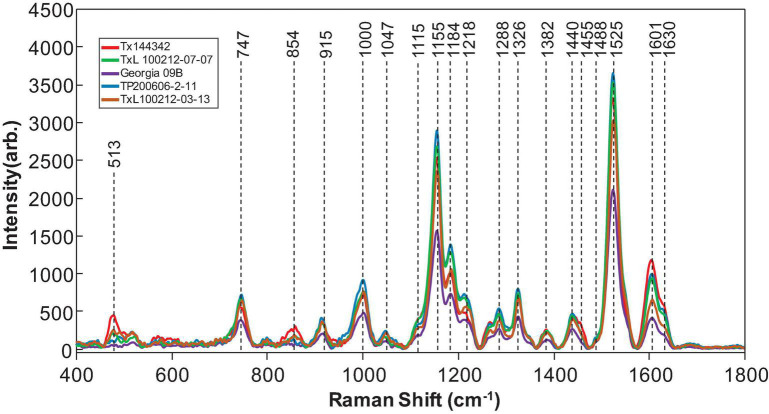
Raman spectra collected from leaves of five representatives of peanut genotypes. In total, 19 peanut genotypes were analyzed.

Raman spectra were collected from 19 different genotypes of peanuts (Figure 1). The spectra exhibited similar profiles with vibrational bands at 480 and 917 cm^–1^, which can be assigned to carbohydrates: 520, 1,048, and 1,115 cm^–1^ to cellulose; 747 and 853 cm^–1^ to pectin; 1,000, 1,155, and 1,526 cm^–1^ to carotenoids; 1,185, 1,606, and 1,632 cm^–1^ to phenylpropanoids (including lignin); 1,660 cm^–1^ to proteins; and 1,682 cm^–1^ to carboxylic acids (Table 2). We also observed vibrational bands at 964, 1,286, 1,327, 1,387, and 1,443 cm^–1^, which can be assigned to aliphatic groups (CH_2_/CH_3_ vibrations) (Farber et al., 2020c).

**TABLE 2:** Vibrational bands and their assignments for spectra collected from peanut leaves.

Band	Vibrational mode	Assignment
513	ν(C-O-C) in plane, symmetric	Cellulose, lignin (Edwards et al., 1997)
747	γ(C–O-H) of COOH	Pectin (Synytsya et al., 2003)
854	ν(C-O-C) in plane, symmetric	Cellulose, lignin (Edwards et al., 1997)
915	ν(C-O-C) in plane, symmetric	Cellulose, lignin (Edwards et al., 1997)
1000	ν_3_ (C-CH_3_ stretching) and phenylalanine	Carotenoids (Tschirner et al., 2009; Kurouski et al., 2015)
1047	ν(C-O) + ν(C-C) + δ(C-O-H)	Cellulose (Almeida et al., 2010)
1115	COH bending	Carotenoids (Adar, 2017; Devitt et al., 2018)
1155	Asym ν(C-C) ring breathing	Carotenoids (Adar, 2017; Devitt et al., 2018)
1184	ν(C-O-H) next to aromatic ring + σ(CH)	Carotenoids (Adar, 2017; Devitt et al., 2018)
1218	δ(C-C-H)	Carotenoids (Adar, 2017; Devitt et al., 2018)
1288	δ(C-C-H)	Aliphatic (Yu et al., 2007)
1326	δCH_2_ bending vibration	Cellulose, lignin (Edwards et al., 1997)
1382	δCH_2_ bending vibration	Aliphatic (Yu et al., 2007)
1440–1555	δ(CH_2_) + δ(CH_3_)	Aliphatic (Yu et al., 2007)
1488	δ(CH_2_) + δ(CH_3_)	Aliphatic (Yu et al., 2007)
1525	-C = C- (in plane)	Carotenoids (Adar, 2017; Devitt et al., 2018)
1601–1630	ν(C-C) aromatic ring + σ(CH)	Phenylpropanoids (Agarwal, 2006; Kang et al., 2016)

We used partial least squares discriminant analysis (PLS-DA) to determine the accuracy of RS in quantitative identification of peanut genotypes based on the spectroscopic signatures collected from their leaflets. As a proof-of-concept study, a subset of six genotypes was chosen that did not contain the same exact maternal and paternal parentage because many of the provided genotypes were closely related sister lines. For example, Tx144370, Tx144485, and Tx144342 are all progeny from the same parents. Because of this, only Tx144342 was selected for modeling since it had the most scans of the three breeding lines and could be more easily used to refine our model. The other five genotypes selected for modeling were TxL100212-02-05, Georgia 09B, Georgia 14N, TP200610-4-8, and Webb. These six genotypes provided the most variety without having breeding lines that were directly related. The results of the model created using these six genotypes indicated that RS averaged about 94% accuracy and ranged from 62% accuracy in some accessions to 100% accuracy in others (see Table 3).

**TABLE 3:** PLS-DA cross-validation confusion matrix of Raman spectra collected from leaves of six different genotypes of peanuts. Results were determined using 50 scans per member sample.

	Predicted genotype							
Genotype sampled	Number of spectra collected per sample	% correct	Tx144342	TxL100212-02-05	Georgia 09B	Georgia 14N	TP200610-4-8	Webb
Tx144342	14	64%	9	0	0	0	0	0
TxL100212-02-05	43	100%	0	43	0	0	0	0
Georgia 09B	38	100%	5	0	38	0	0	0
Georgia 14N	38	97%	0	0	0	37	1	4
TP200610-4-8	49	98%	0	0	0	1	48	1
Webb	13	62%	0	0	0	0	0	8
Total	195	94%						

It was interpreted from the results that the accuracy was correlated with the number of scans suggesting that a relatively higher number of scans such as ∼50 per sample ensures close to 100% accuracy in genotype identification. Expanding upon these results, we created another PLS-DA model except this time only using genotypes with a higher number of scans. The three genotypes chosen were TxL100212-02-05, Georgia 14N, and TP200610-4-8. The results of the model created using these three genotypes and 50 scans per accession sample indicated that RS averaged close to 100% accuracy (99.2%) in genotype identification from peanut leaf scans (see Table 4). These results show that RS can identify peanut plant genotypes from leaf surface scans with very high accuracy.

**TABLE 4:** PLS-DA cross-validation confusion matrix of Raman spectra collected from leaves of three different genotypes of peanuts.

			Predicted genotype		
Genotype tested	Number of spectra collected per sample	% correct	TxL100212-02-05	Georgia 14N	TP200610-4-8
TxL100212-02-05	43	100%	43	0	0
Georgia 14N	38	100%	0	38	1
TP200610-4-8	49	98%	0	0	48
Total	130	99%			

*Results were obtained using 50 scans per sample.*

### Nematode-Resistant vs. Non-nematode-Resistant Genotypes

**FIGURE 2:**
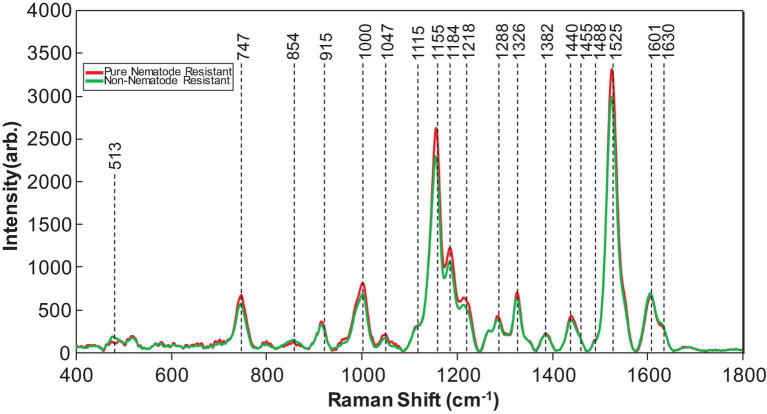
Averaged Raman spectra collected from the leaves of nematode-resistant (referred to as pure nematode-resistant) and susceptible (referred to as non-nematode-resistant) peanut plants.

Of the genotypes provided, both Webb and Georgia 14N were nematode-resistant. The genotypes that are not nematode-resistant were: Tamrun OL11, Georgia 09B, TxL100212-07-07, TxL100212-02-05, TxL100212-05-09, Tx121082, TxL100212-03-13, and TP200606-2-11. These two groups, even though very similar in average spectroscopic signatures (Figure 2), were found to be identified with about 83% (see Table 5) accuracy with PLS-DA modeling. These results show that RS can identify valuable traits such as nematode resistance from leaf surface scans with high accuracy.

**TABLE 5:** PLS-DA cross-validation confusion matrix of Raman spectra collected from leaves of nematode-resistant and susceptible peanut varieties.

	Predicted genotype			
Genotype tested	Number of spectra collected per sample	Correct	Nematode resistant	Nematode susceptible
Nematode resistant	51	92%	47	60
Nematode susceptible	326	81%	4	266
Total	377	83%		

One may wonder about the robustness and reliability of the discussed spectroscopic approach. To answer this question, we excluded nematode-susceptible genotypes from the model and then used that model to verify the accuracy of the prediction of the “left over” genotype (Tables 6, 7). For instance, we built the model using susceptible (Georgia 09B, TxL100212-07-07, TxL100212-02-05, TxL100212-05-09, Tx121082, TxL100212-03-13, and TP200606-2-11) and resistant (Webb and Georgia 14N) peanut varieties with the left over Tarun OL11 variety (Table 6). Next, we used this model to predict the accuracy of identification of Tarun OL11 (Table 7). Our results show that Tamrun OL11 was predicted as susceptible with 84.8% accuracy.

**TABLE 6:** PLS-DA model that is based on susceptible (Georgia 09B, TxL100212-07-07, TxL100212-02-05, TxL100212-05-09, Tx121082, TxL100212-03-13, and TP200606-2-11), and resistant (Webb and Georgia 14N) peanut varieties.

	Predicted genotype			
Genotype tested	Number of spectra collected per sample	Correct	Nematode resistant	Nematode susceptible
Nematode resistant	51	94.1%	46	48
Nematode susceptible	265	84.5%	5	217
Total	316	89.3%		

**TABLE 7:** Prediction results of Tamrun OL11 using PLS-DA model from Table 6.

	Predicted genotype			
Genotype tested	Number of spectra collected per sample	Correct	Nematode resistant	Nematode susceptible
Nematode resistant	0	0	5	0
Nematode susceptible	33	84.8%	28	0

The same validation approach, however, cannot be utilized to demonstrate the robustness of the prediction of nematode-resistant varieties. In the current work, we analyzed only two available-to-date nematode-resistant varieties. If one of them was left out of the model we built and then used for external validation of such a model, the results could have dual interpretation. These results can (i) demonstrate robustness of the spectroscopic approach for identification of nematode resistance and (ii) demonstrate that RS can be used for identification of plant varieties. We previously demonstrated that RS is highly sensitive to plant biochemistry that is drastically different in different peanut varieties (Farber et al., 2020c). This allowed for demonstration of over 80% accurate prediction of such varieties. To overcome this limitation and to demonstrate robustness of the described methodology, we partitioned our data as 60:40; 70:30, and 80:20, where 60, 70, and 80 of the initial data were used to build the model and remaining 40, 30, and 20% were used for external validation (Tables 8–10). All models used the following data prepressing method: SNV, 1st derivative (Sav. Gol), smoothing (Sav. Gol), PQN, normalize, and mean center.

**TABLE 8:** 60:40 prediction and validation model.

	Calibration			
	Predicted as susceptible	Predicted as resistant	Total number	Prediction accuracy,%
Susceptible	109	20	129	84.5
Resistant	3	20	23	87.0
**Validation**				
Susceptible	24	145	169	85.8
Resistant	6	22	28	78.6

**TABLE 9:** 70:30 prediction and validation model.

	Calibration			
	Predicted as susceptible	Predicted as resistant	Total number	Prediction accuracy,%
Susceptible	177	35	212	82.1
Resistant	1	32	33	84.8
**Validation**				
Susceptible	72	14	86	83.7
Resistant	3	15	18	83.3

**TABLE 10:** 80:20 prediction and validation model.

	Calibration			
	Predicted as susceptible	Predicted as resistant	Total number	Prediction accuracy,%
Susceptible	201	37	238	84.5
Resistant	5	37	42	88.1
**Validation**				
Susceptible	54	6	60	90.0
Resistant	2	7	9	77.8

Our results show that similar accuracy of prediction of both susceptible and resistant varieties was obtained upon three various data partitioning approaches. Nevertheless, it should be noted that the reported results of statistical analysis will require at least three additional stages of validation and verification: (1) a larger amount of data for external validation that has to include blind and double-blind spectral assessment strategies; (2) a larger number of nematode-resistant varieties; and (3) elucidation of contribution of environmental factors, such as soil structure, fertilizers, irrigation, and weather conditions.

Critical analysis of chemometric models recently reported by Xu and Goodacre showed that (i) the size of the dataset and (ii) choice of the data splitting methods were critical for model performance (Xu and Goodacre, 2018). Xu and Goodacre determined that datasets with less than 30 spectra were unlikely to be suitable for the development of robust and reliable methods. The researchers also found that model performance was also very sensitive to the choice of a data splitting method used to partition the training data into training and validation sets. The reported results of the current study are based on 51 spectra collected for resistant and 326 spectra collected from susceptible varieties. We envision that although different spectral partitioning strategies provided similar outcomes, collection of 300–1,000 spectra from the resistant varieties will be required in the future to enable external validation of the reported models. We expect that such a large dataset will be sufficient to fully validate the robustness of the reported proof-of-principle spectroscopic approach in the current study. We also anticipate that development of novel nematode-resistant varieties, which is currently in progress in our laboratory, will allow for additional generalization of the reported spectroscopic approach. Finally, we expect to reproduce the reported studies in several different geographic locations in the U.S. to investigate the extent to which environmental factors alter spectroscopic signatures of nematode-resistant and susceptible peanut varieties.

### Nutrient Content Analysis

From analyzing Figure 2, the vibrational bands at 1,155–1,218 cm^–1^ that are associated with carotenoids had a higher intensity in nematode-resistant varieties than nematode-susceptible varieties (Payne and Kurouski, 2011). Likewise, the vibrational band at 1,525 cm^–1^ that is also associated with carotenoids was also more intense in nematode-resistant varieties. However, the vibrational band at 1,606 cm^–1^, associated with phenylpropanoids, was much more intense in nematode-susceptible varieties (Payne and Kurouski, 2011; Farber et al., 2019b, 2020a; Sanchez et al., 2020a). These data suggest that nematode-resistant peanut plants are higher in carotenoid content than nematode-susceptible peanut plants.

It also should be noted that RS analysis costs are much lower than traditional genomic analysis because there is no additional cost incurred on a per sample basis for the phenotyping of plants. In addition, the hand-held nature of Raman spectrometers enables their use for on-site analysis of plants which further lowers overall costs and increases efficiency. Most currently available hand-held RS instruments have incorporated computers that allow for chemometric analysis of spectra immediately upon acquisition. Thus, integration of the spectroscopic libraries of germplasm into the hand-held spectrometer units eliminate the need to transfer the collected spectra for later analyses at a different location. As trait libraries are expanded and developed, researchers, seed companies, consultants, and even growers can obtain results almost instantaneously which will allow them to make almost real-time decisions in the field from the small screen of the RS spectrometers.

## Conclusion

Our results demonstrate that RS can be used for highly accurate identification of peanut genotypes. We showed that with a proper number of scans, PLS-DA can be used to build a model that can identify specific peanut genotypes by leaf scans with close to 100% accuracy. Additionally, we demonstrated that RS can be potentially used for indication of valued traits such as nematode resistance by leaf scans with approximately 83% accuracy. The ability to distinguish cultivars will allow peanut breeders, shellers, and processors to maintain high purity levels at all levels of the value chain. Finally, we showed how analysis of leaf spectra may give insight into peanut plant biochemistry which could be used as a possible direction to further the resistance mechanism that is present in nematode-resistant germplasm. In addition, the fast, portable nature of RS allows for researchers, consultants, and growers to begin to implement the use of RS for digital farming/precision agriculture. With tools like RS at their disposal, they can better selectively breed and manage the valued traits, and maximize production.

## Data Availability Statement

The raw data supporting the conclusions of this article will be made available by the authors, without undue reservation.

## Author Contributions

WP performed Raman measurements, data curation, statistical analysis, analyzed spectroscopic data, discussed results, and wrote the manuscript. JC provided plant material and supervision, discussed results, and wrote the manuscript. DK provided supervision, discussed results, wrote the manuscript, and acquired funding. All authors approved the final version of this manuscript.

## Conflict of Interest

The authors declare that the research was conducted in the absence of any commercial or financial relationships that could be construed as a potential conflict of interest.

## Publisher’s Note

All claims expressed in this article are solely those of the authors and do not necessarily represent those of their affiliated organizations, or those of the publisher, the editors and the reviewers. Any product that may be evaluated in this article, or claim that may be made by its manufacturer, is not guaranteed or endorsed by the publisher.

## References

[B1] Adar F. (2017). Carotenoids - their resonance raman spectra and how they can be helpful in characterizing a number of biological systems. *Spectroscopy* 32 12–20.

[B2] Agarwal U. P. (2006). Raman imaging to investigate ultrastructure and composition of plant cell walls: distribution of lignin and cellulose in black spruce wood (*Picea mariana*). *Planta* 224 1141–1153. 10.1007/s00425-006-0295-z 16761135

[B3] Almeida M. R. Alves R. S. Nascimbem L. B. Stephani R. Poppi R. J. De Oliveira L. F. (2010). Determination of amylose content in starch using Raman spectroscopy and multivariate calibration analysis. *Anal. Bioanal. Chem.* 397 2693–2701. 10.1007/s00216-010-3566-2 20213166

[B4] Branch W. D. Brenneman T. B. (2014). Registration of ‘Georgia-14N’ Peanut. *J. Plant Regist.* 9 159–161.

[B5] Burow G. Chopra R. Hughes H. Burke J. Xin Z. (2019). Marker assisted selection in sorghum using KASP assay for the detection of single nucleotide polymorphism/insertion deletion. *Methods Mol. Biol.* 1931 75–84. 10.1007/978-1-4939-9039-9_6 30652284

[B6] Burow M. D. Burow S. C. Leal-Bertioli M. Simpson C. E. Ozias-Akins P. Chu Y. (2013). “Chapter 8 Marker-Assisted Selection for Biotic Stress Resistance in Peanut,” in *Translational Genomics for Crop Breeding Volume I*, eds Varshney R. K. Tuberosa R. (Hoboken, New Jersey: John Wiley and Sons Inc).

[B7] Burow M. D. Simpson C. E. Paterson A. H. Starr J. L. (1996). Identification of peanut (*Arachis hypogaea*) RAPD markers diagnostic of root-knot nematode (*Meloidogyne arenaria* (Neal) Chitwood) resistance. *Mol. Breed.* 2 307–319.

[B8] Cason J. M. Simpson C. E. Rooney W. L. Brady J. A. (2020). Drought-tolerant transcription factors identified in *Arachis* dardani and *Arachis ipaënsis*. *Agrosyst. Geosci. Environ.* 3:e20069.

[B9] Chu Y. Wu L. Holbrook C. C. Tillman B. L. Person G. Ozias-Akins P. (2011). Marker-Assisted Selection to Pyramid Nematode Resistance and the High Oleic Trait in Peanut. *Plant Genome* 4 110–117.

[B10] Devitt G. Howard K. Mudher A. Mahajan S. (2018). Raman spectroscopy: an emerging tool in neurodegenerative disease research and diagnosis. *ACS Chem. Neurosci.* 9 404–420. 10.1021/acschemneuro.7b00413 29308873

[B11] Dong W. Holbrook C. C. Timper P. Brenneman T. B. Mullinix B. G. (2007). Comparison of Methods for Assessing Resistance to *Meloidogyne arenaria* in Peanut. *J. Nematol.* 39 169–175. 19259486 PMC2586494

[B12] Edwards H. G. Farwell D. W. Webster D. (1997). FT Raman microscopy of untreated natural plant fibres. *Spectrochim. Acta A Mol. Biomol. Spectrosc.* 53 2383–2392. 10.1016/s1386-1425(97)00178-9 9477578

[B13] Egging V. Nguyen J. Kurouski D. (2018). Detection and identification of fungal infections in intact wheat and sorghum hrain using a hand-held Raman spectrometer. *Anal. Chem.* 90 8616–8621. 10.1021/acs.analchem.8b01863 29898358

[B14] Eriksson L. Byrne T. Johansson E. Trygg J. Vikstrom C. (2013). *Multi- and Megavariate Data Analysis Basic Principles and Applications.* Malmö, Sweden: Umetrics.

[B15] Farber C. Bryan R. Paetzold L. Rush C. Kurouski D. (2020a). Non-Invasive Characterization of Single-, Double- and Triple-Viral Diseases of Wheat With a Hand-Held Raman Spectrometer. *Front. Plant Sci.* 11:01300. 10.3389/fpls.2020.01300 33013951 PMC7495046

[B16] Farber C. Sanchez L. Kurouski D. (2020b). Confirmatory Non-Invasive and Non-Destructive Identification of Poison Ivy Using A Hand-Held Raman Spectrometer. *RCS Adv.* 10 21530–21534. 10.1039/d0ra03697h PMC9054379 35518747

[B17] Farber C. Sanchez L. Rizevsky S. Ermolenkov A. Mccutchen B. Cason J. (2020c). Raman Spectroscopy Enables Non-Invasive Identification of Peanut Genotypes and Value-Added Traits. *Sci. Rep.* 10:7730. 10.1038/s41598-020-64730-w 32382086 PMC7206150

[B18] Farber C. Kurouski D. (2018). Detection and Identification of Plant Pathogens on Maize Kernels with a Hand-Held Raman Spectrometer. *Anal. Chem.* 90 3009–3012. 10.1021/acs.analchem.8b00222 29461798

[B19] Farber C. Mahnke M. Sanchez L. Kurouski D. (2019a). Advanced Spectroscopic Techniques for Plant Disease Diagnostics. A Review. *Trends Analyt. Chem.* 118 43–49. 10.1016/j.trac.2019.05.022

[B20] Farber C. Shires M. Ong K. Byrne D. Kurouski D. (2019b). Raman spectroscopy as an early detection tool for rose rosette infection. *Planta* 250 1247–1254. 10.1007/s00425-019-03216-0 31222494

[B21] Food and Agriculture Organization of the United Nations (2009). *How to Feed the World 2050.* Rome: Food and Agriculture Organization.

[B22] Hayes B. J. Daetwyler H. D. Bowman P. Moser G. Tier B. Crump R. (2009). Accuracy of genomic selection: comparing theory and results. *Proc. Assoc. Advmt. Anim. Breed. Genet.* 18 34–37.

[B23] Heffner E. L. Sorrells M. E. Jannink J. L. (2009). Genomic selection for crop improvement. *Crop Sci.* 49 1–2. 10.1007/978-3-319-63170-7_1

[B24] Holbrook C. C. Ozias-Akins P. Chu Y. Culbreath A. K. Kvien C. K. Brenneman T. B. (2017). Registration of ‘TifNV-High O/L’ Peanut. *J. Plant Regist.* 11 228–230. 10.3198/jpr2016.10.0059crc

[B25] Holbrook C. C. Timper P. Culbreath A. K. Kvien C. K. (2008). Registration on ‘Tifguard’ Peanut. *J. Plant Regist.* 2 92–94. 10.3198/jpr2007.12.0662crc

[B26] Kang L. Wang K. Li X. Zou B. (2016). High pressure structural investigation of benzoic acid: raman spectroscopy and x-ray diffraction. *J. Phys. Chem. C* 120 14758–14766.

[B27] Kochert G. D. Halward T. M. Branch W. D. Simpson C. E. (1991). RFLP variability in peanut cultivars and wild species. *Theor. Appl. Genet.* 81 565–570. 10.1007/BF00226719 24221368

[B28] Krapovickas A. Gregory W. C. (1994). Taxonomia del genero *Arachis* (*Leguminosae*). *Bonplandia* 8 1–186. 10.30972/bon.160158

[B29] Krapovickas A. Gregory W. C. (2007). Taxonomy of the genus *Arachis* (Leguminosae), Williams D. E. Simpson C. E. *Bonplandia* 16(Supll.), 1–205.

[B30] Kurouski D. Van Duyne R. P. Lednev I. K. (2015). Exploring the structure and formation mechanism of amyloid fibrils by Raman spectroscopy: a review. *Analyst* 140 4967–4980. 10.1039/c5an00342c 26042229

[B31] Morey R. Ermolenkov A. Payne W. Z. Scheuring D. C. Koym J. W. Vales M. I. (2020). Non-invasive identification of potato varieties and prediction of the origin of tuber cultivation using spatially offset Raman spectroscopy. *Anal. Bioanal. Chem.* 412 4585–4594. 10.1007/s00216-020-02706-5 32451641

[B32] NPB (2020). *History of peanuts and peanut butter.* Available online at: http://nationalpeanutboard.org/peanut-info/history-peanuts-peanut-butter.htm (accessed December 1, 202)

[B33] Payne W. Z. Kurouski D. (2011). Raman-based diagnostics of biotic and abiotic stresses in plants. A review. *Front. Plant Sci.* 11:616672. 10.3389/fpls.2020.616672 33552109 PMC7854695

[B34] Ravelombola W. S. Qin J. Shi A. Nice L. Bao Y. Lorenz A. (2019). Genome-wide association study and genomic selection for soybean chlorophyll content associated with soybean cyst nematode tolerance. *BMC Genomics* 20:904. 10.1186/s12864-019-6275-z 31775625 PMC6882315

[B35] Ravelombola W. S. Qin J. Shi A. Nice L. Bao Y. Lorenz A. (2020). Genome-wide association study and genomic selection for tolerance of soybean biomass to soybean cyst nematode infestation. *PLoS One* 17:e0235089. 10.1371/journal.pone.0235089 32673346 PMC7365597

[B36] Rich J. Tillman B. (2009). *Root-Knot Nematode Resistance in Peanut1. UF/IFAS Extension ENY057.* Florida: University of Florida.

[B37] Sanchez L. Ermolenkov A. Tang X. T. Tamborindeguy C. Kurouski D. (2020a). Non-invasive diagnostics of Liberibacter disease on tomatoes using a hand-held Raman spectrometer. *Planta* 251:64. 10.1007/s00425-020-03359-5 32048047

[B38] Sanchez L. Pant S. Mandadi K. Kurouski D. (2020b). Raman Spectroscopy vs Quantitative Polymerase Chain Reaction In Early Stage Huanglongbing Diagnostics. *Sci. Rep.* 10:10101. 10.1038/s41598-020-67148-6 32572139 PMC7308309

[B39] Sanchez L. Farber C. Lei J. Zhu-Salzman K. Kurouski D. (2019a). Noninvasive and Nondestructive Detection of Cowpea Bruchid within Cowpea Seeds with a Hand-Held Raman Spectrometer. *Anal. Chem.* 91 1733–1737. 10.1021/acs.analchem.8b05555 30620572

[B40] Sanchez L. Pant S. Irey M. S. Mandadi K. Kurouski D. (2019b). Detection and Identification of Canker and Blight on Orange Trees Using a Hand-Held Raman Spectrometer. *J. Raman Spectrosc.* 50 1875–1880.

[B41] Simpson C. E. Starr J. L. (2001). Registration of ‘COAN’ peanut. *Crop Sci.* 41:918. 10.2135/cropsci2001.413918x 34798789

[B42] Simpson C. E. Starr J. L. Church G. T. Baring M. R. Burow M. D. (2013). Registration of ‘Webb’ Peanut. *J. Plant Regist.* 7:265. 10.1111/cea.13816 33386634

[B43] Simpson C. E. Starr J. L. Church G. T. Burow M. D. Paterson A. H. (2003). Registration of ‘NemaTAM’ Peanut. *Crop Sci.* 43:1561. 10.2135/cropsci2003.1561 34798789

[B44] Singh A. K. Simpson C. E. (1994). “Biosystematics and genetic resources,” in *The Groundnut Crop: a Scientific Basis for Improvement*, ed. Smartt J. (London: Chapman & Hall), 96–137. 10.1007/978-94-011-0733-4_4

[B45] Synytsya A. Čopíková J. Matějka P. Machovič V. (2003). Fourier transform Raman and infrared spectroscopy of pectins. *Carbohydr. Polym.* 54 97–106.

[B46] Timper P. Holbrook C. C. Xue H. Q. (2000). Expression of nematode resistance in plant introduction of *Arachis hypogaea*. *Peanut Sci.* 27 78–82.

[B47] Tirumalaraja S. V. Jain M. Gallo M. (2011). Differential gene expression in roots of nematode-resistant and -susceptible peanut (*Arachis hypogaea*) cultivars in response to early stages of peanut root-knot nematode (*Meloidogyne arenaria*) parasitization. *J. Plant Physiol.* 168 481–492. 10.1016/j.jplph.2010.08.006 20863592

[B48] Tschirner N. Brose K. Schenderlein M. Zouni A. Schlodder E. Mroginski M. A. (2009). The anomaly of the $\nu$1-resonance Raman band of bβ-carotene in solution and in photosystem I and II. *Phys. Status Solidi* 246 2790–2793.

[B49] USDA-NASS (2019). *USDA-NASS. 2019. Crop Production Statistics 2019 Summary.* Available Online at: www.nass.usda.gov/Statistics_by_Subject (accessed May 3, 2020)

[B50] Xu Y. Goodacre R. (2018). On Splitting Training and Validation Set: a Comparative Study of Cross-Validation, Bootstrap and Systematic Sampling for Estimating the Generalization Performance of Supervised Learning. *J. Anal. Test.* 2 249–262. 10.1007/s41664-018-0068-2 30842888 PMC6373628

[B51] Yu M. M. Schulze H. G. Jetter R. Blades M. W. Turner R. F. (2007). Raman microspectroscopic analysis of triterpenoids found in plant cuticles. *Appl. Spectrosc.* 61 32–37. 10.1366/000370207779701352 17311714

